# Association of anemia and iron parameters with mortality among prevalent peritoneal dialysis patients in Taiwan: the AIM-PD study

**DOI:** 10.1038/s41598-022-05200-3

**Published:** 2022-01-24

**Authors:** Ko-Lin Kuo, Jia-Sin Liu, Ming-Huang Lin, Chih-Cheng Hsu, Der-Cherng Tarng, the Taiwan Society of Nephrology Renal Registry Data System Research Group, Szu-Chun Hung, Ko-Lin Kuo, Jia-Sin Liu, Chih-Cheng Hsu, Ming-Huang Lin, Der-Cherng Tarng, Wei-Cheng Tseng, Ming-Tsun Tsai, Shuo-Ming Ou, Chih-Yu Yang, Yao-Ping Lin, Yi-Sheng Lin, Chia-Lin Wu, Tung-Po Hung

**Affiliations:** 1 Division of Nephrology, Taipei Tzu Chi Hospital, Buddhist Tzu Chi Medical Foundation, Taipei, Taiwan; 2 School of Medicine, Tzu Chi University, Hualien, Taiwan; 3 School of Post-Baccalaureate Chinese Medicine, Tzu Chi University, Hualien, Taiwan; 4 Department of Public Health, Kaohsiung Medical University, Kaohsiung, Taiwan; 5 Institute of Population Health Sciences, National Health Research Institutes, 35, Keyan Road, Zhunan Town, Miaoli County 35053 Taiwan; 6 Department of Health Services Administration, China Medical University, Taichung, Taiwan; 7 Department of Family Medicine, Min-Sheng General Hospital, Taoyüan, Taiwan; 8 Institute of Clinical Medicine, National Yang Ming Chiao Tung University, Taipei, Taiwan; 9 Center for Intelligent Drug Systems and Smart Bio-Devices (IDS2B), Hsinchu, Taiwan; 10 Department and Institute of Physiology, National Yang Ming Chiao Tung University, 201, Section 2, Shih-Pai Road, Taipei, 11217 Taiwan; 11 Division of Nephrology, Department of Medicine, Taipei Veterans General Hospital, Taipei, Taiwan; 12 Division of Nephrology, Taipei City Hospital, Taipei, Taiwan; 13 Division of Nephrology, Changhua Christian Hospital, Changhua, Taiwan; 14 Division of Nephrology, Wei Gong Memorial Hospital, Toufen, Miaoli Taiwan

**Keywords:** Medical research, Nephrology

## Abstract

In 1996, the National Health Insurance Administration of Taiwan applied a restrictive reimbursement criteria for erythropoiesis-stimulating agents (ESAs) use in patients with chronic kidney disease. The maximal ESAs dosage allowed by insurance is capped at 20,000 U of epoetin per month. Nephrologists avoided the use of high ESA dosages to achieve a hemoglobin level of 10–11 g/dL using iron supplementation. We assessed the association of anemia and iron parameters with mortality among peritoneal dialysis (AIM-PD) patients. A retrospective cohort study was conducted based on the Taiwan Renal Registry Data System. From January 1, 2000 to December 31, 2008, we enrolled 4356 well-nourished PD patients who were older than 20 years and had been receiving PD for more than 12 months. All patients were divided into subgroups according to different hemoglobin, ferritin and transferrin saturation (TSAT) values. Patients were followed until death or December 31, 2008. In a median 2.9-year study period, 694 (15.9%) patients died. By multivariate adjustment, a hemoglobin level lower than 10 g/dL was significantly associated with a higher risk for all-cause and cardiovascular deaths. Moreover, a serum ferritin level higher than 800 ng/mL was associated with a higher risk for all-cause deaths, and a TSAT value between 20 and 50% was associated with the lowest all-cause mortality. In conclusions, we recommend avoiding a low hemoglobin level and a serum ferritin level of more than 800 ng/mL and maintaining a TSAT value between 20 and 50%, as these conditions were associated with lower risks of all-cause mortality in the AIM-PD study.

## Introduction

Anemia is frequently encountered in chronic kidney disease (CKD) and is associated with adverse cardiovascular (CV) outcomes in CKD patients^1^. Correcting anemia usually requires the administration of erythropoiesis-stimulating agents (ESAs). However, the administration of ESAs to normalize hemoglobin levels was associated with an increased risk of cardiovascular events and even death^2–5^. Moreover, use of iron supplementation along with ESAs is pivotal for the optimal anemia management in CKD patients^6^. Iron therapy could reduce ESA requirements and increase hemoglobin levels^7^.

Taiwan was the first country in the world to implement a bundled payment system for CKD patients who underwent hemodialysis (HD) and peritoneal dialysis (PD) due to economic concerns^8,9^. The strategy of anemia management for CKD patients is different from that in other parts of the world. Since 1996, the National Health Insurance Administration of Taiwan has applied more restrictive reimbursement criteria for ESAs use in patients with CKD stage 5. According to this regulation, ESAs could be initiated when non-dialysis CKD patients have a serum creatinine level > 6 mg/dL and a hematocrit < 28% in order to maintain a hematocrit level not exceeding 30%. The monthly maximum dosage allowed by insurance is capped at 20,000 U of epoetin-α or β or other ESAs with the same equal conversion dose. The target hemoglobin range and dosage limitation for ESAs were the same for HD and PD patients^8,9^. Meanwhile, intravenous (IV) iron supplementation has been encouraged in Taiwan since 1996, when nephrology experts reached a consensus regarding the diagnostic criteria for iron deficiency by serum ferritin < 300 ng/mL and/or transferrin saturation (TSAT) < 30%. Thereafter, nephrologists in Taiwan avoided the use of disproportionately high dosages of ESAs to achieve a hemoglobin level of 10–11 g/dL by increasing iron supplementation^8,9^. The influence of these policies has been shown in HD patients^9^, but the impact in PD patients remains unknown. Using data from the Taiwan Renal Registry Data System (TWRDS)^10^, we aimed to evaluate the association of **A**nemia and **I**ron parameters with **M**ortality among prevalent **P**eritoneal **D**ialysis patients (AIM-PD) in Taiwan. In the AIM-PD study, we assessed the associations of optimal hemoglobin, serum ferritin and TSAT levels with the less mortality in PD patients under the implementation of Taiwan bundled payment system for ESA use.

## Results

### Patient characteristics

**Figure 1:**
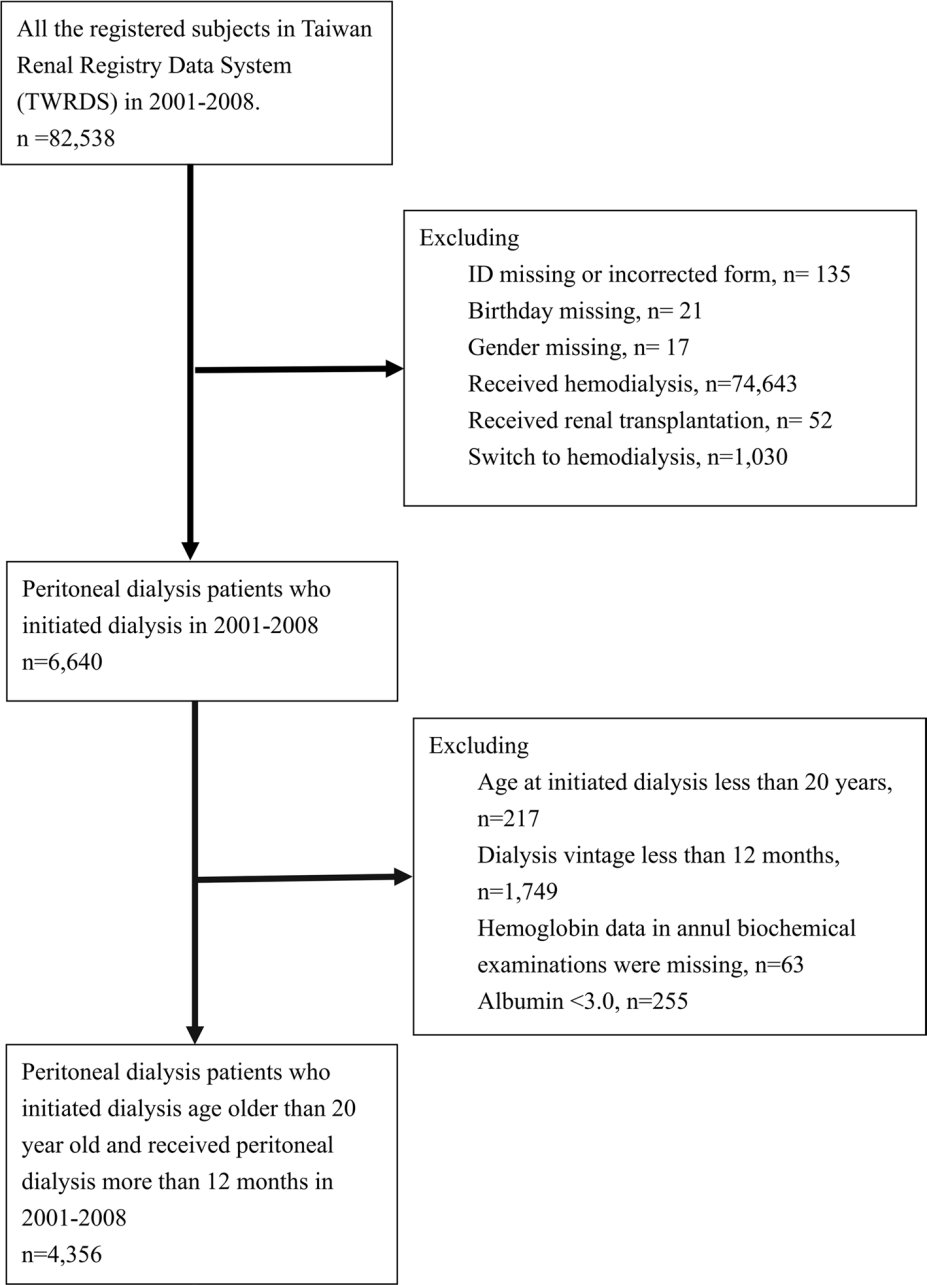
Flowchart of patient selection.

Figure 1 shows the flowchart of patients selected from the TWRDS from 2001 to 2008. Ultimately, 4356 stable PD patients were enrolled for analysis. All patients were divided into four groups according to hemoglobin level (< 10, 10–10.9, 11–11.9, and ≥ 12 g/dL) (Table 1), ferritin level (< 300, 300–499, 500–799, and ≥ 800 ng/mL) (Table 2) and TSAT value (< 20, 20–29, 30–49, and ≥ 50%) (Table 3). The stratification of ferritin and TSAT was mainly based on our previous studies and the recommendation of Taiwan Society of Nephrology^8,9^. The data revealed statistically significant differences among the groups in all measured parameters (Tables 1, 2, 3). Table 1 shows that comparing PD patients with a hemoglobin level greater than 10 g/dL, those with a hemoglobin level < 10 g/dL were younger, tended to be female, and had higher serum ferritin levels, mean administered ESA doses and IV iron administration rates. Additionally, Table 2 shows that comparing PD patients with a ferritin level less than 800 ng/dL, those with a ferritin level ≥ 800 ng/dL were older, tended to be female and had higher mean administered ESA doses but lower levels of serum albumin and IV iron administration rates. Finally, in Table 3, comparing PD patients with TSAT values of 30–50%, those with TSAT values ≥ 50% were older, tended to be male, and had higher hypoalbuminemia and dialysis inadequacy rates. While compared with the latest PD data in Nov. 2020 in DOPPS^11^, our PD cohort are more likely to be anemic and had lower serum ferritin, but similar TSAT level (Supplemental Table S1).

**Table 1:** Time-averaged characteristics of peritoneal dialysis patients stratified by four hemoglobin concentration groups.

Characteristics	Hemoglobin (g/dL)				
	< 10	10–10.9	11–11.9	≥ 12	*p* value
N	2148	1445	598	165	
Age (years)	50.0 (14.6)	53.0 (14.4)	54.3 (14.8)	53.6 (15.0)	< 0.0001
**Age group**					
20–39 years, n (%)	537 (25.0)	258 (17.9)	103 (17.2)	30 (18.2)	< 0.0001
40–64 years, n (%)	1222 (56.9)	863 (59.7)	347 (58.0)	94 (57.0)	0.4043
65–74 years, n (%)	274 (12.8)	215 (14.9)	89 (14.9)	24 (14.6)	0.2570
75 + years, n (%)	115 (5.4)	109 (7.5)	59 (9.9)	17 (10.3)	0.0002
**Gender**					
Female, n (%)	1368 (63.7)	791 (54.7)	259 (43.2)	51 (30.9)	< 0.0001
Diabetes, n (%)	491 (22.9)	334 (23.1)	142 (23.8)	49 (29.7)	0.2506
Hypertension, n (%)	1539 (71.7)	1044 (72.3)	433 (72.4)	116 (70.3)	0.9320
Weekly Kt/V	2.1 (0.5)	2.1 (0.5)	2.1 (0.5)	1.9 (0.7)	< 0.0001
Weekly CCr (L/week/1.73 m^2^)	57.3 (16.0)	60.8 (17.6)	63.2 (20.6)	59.4 (28.0)	< 0.0001
eGFR at the start of dialysis (MDRD)	4.7 (2.1)	5.2 (1.9)	5.7 (2.4)	6.4 (4.0)	< 0.0001
WBC (× 10^3^/μl)	5.9 (2.5)	6.1 (2.4)	6.1 (2.3)	6.1 (2.5)	0.0197
Hemoglobin (g/dL)	9.1 (0.8)	10.5 (0.3)	11.4 (0.3)	12.6 (0.9)	< 0.0001
Ferritin (ng/dL)	496.8 (406.7)	382.7 (316.8)	372.5 (333.0)	306.3 (254.5)	< 0.0001
TSAT (%)	30.5 (11.5)	30.8 (9.7)	32.3 (10.4)	30.4 (10.4)	0.0061
Serum calcium (mg/dL)	9.0 (2.0)	9.0 (1.8)	8.9 (2.0)	8.7 (2.5)	0.1551
Serum phosphate (mg/dL)	5.3 (1.2)	5.0 (0.9)	4.8 (0.9)	4.7 (1.1)	< 0.0001
Alkaline phosphatase (U/L)	108.0 (57.3)	106.3 (57.8)	107.0 (55.5)	105.3 (56.8)	0.8074
Intact-PTH (pg/L)	307.6 (264.4)	277.3 (225.6)	252.7 (235.3)	237.0 (205.6)	< 0.0001
Uric acid (mg/dL)	7.1 (1.3)	6.9 (1.2)	6.9 (1.4)	7.1 (1.7)	< 0.0001
Cholesterol (mg/dL)	199.2 (38.3)	200.6 (35.8)	195.1 (37.2)	191.6 (39.0)	0.0015
Triglyceride (mg/dL)	196.4 (122.6)	172.7 (99.0)	168.3 (83.0)	192.1 (103.0)	< 0.0001
nPCR	0.1 (0.3)	0.1 (0.3)	0.1 (0.2)	0.1 (0.3)	0.0115
Albumin (g/dL)	3.8 (1.1)	3.8 (0.3)	3.8 (0.4)	3.9 (0.4)	0.2841
ESA dose, (U/month)	17,647.8 (7156.9)	16,314.5 (6183.9)	14,498.0 (8317.9)	11,331.8 (8364.6)	< 0.0001
IV Iron, n (%)	1088 (50.7)	704 (48.7)	236 (39.5)	53 (32.1)	< 0.0001

ESA: erythropoiesis-stimulating agent; eGFR: estimated glomerular filtration rate; IV: intravenous; nPCR: normalized protein catabolic rate; PTH, parathyroid hormone; TSAT: transferrin saturation; WBC: white blood cell counts. All values are expressed as the mean (SD) unless otherwise specified.

**Table 2:** Time-averaged characteristics of peritoneal dialysis patients stratified by four serum ferritin concentration groups.

Characteristics	Ferritin (ng/mL)				
	< 300	300–499	500–799	≥ 800	*p* value
n	1919	1134	778	525	
Age (years)	49.6 (14.4)	52.0 (14.2)	54.0 (14.5)	55.4 (15.8)	< 0.0001
**Age group**					
20–39 years, n (%)	479 (24.9)	217 (19.1)	127 (16.3)	105 (20.0)	< 0.0001
40–64 years, n (%)	1119 (58.3)	698 (61.6)	460 (59.1)	249 (47.4)	< 0.0001
65–74 years, n (%)	219 (11.4)	144 (12.7)	127 (16.3)	112 (21.3)	< 0.0001
75 + years, n (%)	102 (5.3)	75 (6.6)	64 (8.2)	59 (11.2)	< 0.0001
**Gender**					
Female, n (%)	1156 (60.2)	607 (53.5)	391 (50.2)	315 (60.0)	< 0.0001
Diabetes, n (%)	419 (21.8)	260 (22.9)	193 (24.8)	144 (27.4)	0.0387
Hypertension, n (%)	1386 (72.2)	864 (76.2)	551 (70.8)	331 (63.1)	< 0.0001
Weekly Kt/V	2.1 (0.5)	2.1 (0.4)	2.0 (0.5)	2.0 (0.5)	< 0.0001
Weekly CCr (L/week/1.73 m^2^)	60.0 (18.7)	60.6 (16.6)	58.7 (17.6)	54.8 (17.7)	< 0.0001
eGFR at the start of dialysis (MDRD)	5.0 (2.4)	4.9 (1.7)	5.1 (2.1)	5.4 (2.8)	0.0007
WBC (× 10^3^/μl)	5.8 (2.4)	6.1 (2.3)	6.3 (2.3)	6.2 (2.7)	< 0.0001
Hemoglobin (g/dL)	10.2 (1.1)	10.0 (1.1)	9.8 (1.1)	9.4 (1.3)	< 0.0001
Ferritin (ng/dL)	170.9 (76.7)	388.4 (56.1)	622.5 (83.2)	1220.4 (405.8)	< 0.0001
TSAT (%)	27.4 (9.1)	30.7 (8.2)	33.0 (9.2)	40.7 (15.2)	< 0.0001
Serum calcium (mg/dL)	8.9 (2.0)	9.1 (1.8)	9.0 (2.0)	8.9 (2.0)	0.3013
Serum phosphate (mg/dL)	5.2 (1.0)	5.2 (1.0)	5.1 (1.0)	5.1 (1.2)	0.0250
Alkaline phosphatase (U/L)	101.9 (55.3)	109.6 (58.4)	110.3 (56.9)	116.7 (60.2)	< 0.0001
Intact-PTH (pg/L)	295.9 (250.9)	292.8 (239.8)	278.8 (256.3)	256.8 (231.5)	0.0080
Uric acid (mg/dL)	7.0 (1.3)	7.0 (1.2)	7.1 (1.2)	7.0 (1.4)	0.2876
Cholesterol (mg/dL)	199.7 (38.2)	198.8 (35.1)	196.5 (36.3)	199.0 (40.8)	0.2496
Triglyceride (mg/dL)	168.3 (98.1)	184.0 (109.2)	196.0 (110.3)	227.6 (137.6)	< 0.0001
nPCR	0.1 (0.3)	0.1 (0.3)	0.1 (0.3)	0.1 (0.3)	0.0030
Albumin (mg/dL)	3.8 (0.3)	3.9 (1.5)	3.8 (0.4)	3.7 (0.4)	0.0021
ESA dose, (U/month)	16,124.0 (8277.8)	16,803.5 (5777.1)	16,724.4 (6973.2)	17,167.0 (6176.0)	0.0064
Iron IV, n (%)	1039 (54.1)	620 (54.7)	301 (38.7)	121 (23.1)	< 0.0001

ESA: erythropoiesis-stimulating agent; eGFR: estimated glomerular filtration rate; IV: intravenous; nPCR: normalized protein catabolic rate; PTH, parathyroid hormone; TSAT: transferrin saturation; WBC: white blood cell counts. All values are expressed as the mean (SD) unless otherwise specified.

**Table 3:** Time-averaged characteristics of peritoneal dialysis patients stratified by four TSAT percentage groups.

Characteristics	TSAT (%)				
	< 20	20–29	30–49	≥ 50	*p* value
n	436	1892	1828	200	
Age (years)	50.8 (13.9)	52.0 (13.8)	51.3 (15.6)	54.5 (16.2)	0.0117
**Age group**					
20–39 years, n (%)	95 (21.8)	359 (19.0)	436 (23.9)	38 (19.0)	0.0031
40–64 years, n (%)	267 (61.2)	1173 (62.0)	982 (53.7)	104 (52.0)	< 0.0001
65–74 years, n (%)	48 (11.0)	253 (13.4)	265 (14.5)	36 (18.0)	0.0769
75 + years, n (%)	26 (6.0)	107 (5.7)	145 (7.9)	22 (11.0)	0.0038
**Gender**					
Female, n (%)	266 (61.0)	1094 (57.8)	1006 (55.0)	103 (51.5)	0.0361
Diabetes, n (%)	154 (35.3)	486 (25.7)	332 (18.2)	44 (22.0)	< 0.0001
Hypertension, n (%)	316 (72.5)	1411 (74.6)	1311 (71.7)	94 (47.0)	< 0.0001
Weekly Kt/V	2.0 (0.7)	2.1 (0.4)	2.1 (0.4)	1.9 (0.7)	< 0.0001
Weekly CCr (L/week/1.73 m^2^)	57.9 (22.7)	59.8 (16.8)	59.7 (17.0)	53.8 (23.0)	< 0.0001
eGFR at the start of dialysis (MDRD)	5.3 (3.7)	4.9 (2.0)	5.0 (1.6)	5.9 (4.1)	< 0.0001
WBC (× 10^3^/μl)	6.5 (2.7)	6.2 (2.4)	5.8 (2.3)	5.8 (2.7)	< 0.0001
Hemoglobin (g/dL)	9.7 (1.3)	10.0 (1.1)	10.1 (1.1)	9.7 (1.5)	< 0.0001
Ferritin (ng/dL)	239.9 (225.5)	352.0 (266.9)	501.4 (364.1)	1031.2 (657.8)	< 0.0001
TSAT (%)	16.4 (4.7)	25.5 (2.8)	36.4 (4.9)	62.4 (12.5)	< 0.0001
Serum calcium (mg/dL)	8.9 (2.1)	9.0 (1.9)	8.9 (1.9)	8.6 (2.3)	0.0133
Serum phosphate (mg/dL)	5.5 (1.2)	5.3 (1.0)	5.0 (1.0)	4.9 (1.2)	< 0.0001
Alkaline phosphatase (U/L)	110.7 (63.6)	108.1 (57.5)	104.6 (54.7)	115.1 (61.9)	0.0199
Intact-PTH (pg/L)	286.1 (263.6)	293.7 (252.2)	284.1 (232.6)	260.1 (284.5)	0.2584
Uric acid (mg/dL)	7.2 (1.6)	7.1 (1.2)	6.9 (1.2)	6.9 (1.3)	< 0.0001
Cholesterol (mg/dL)	198.1 (49.9)	202.0 (35.2)	196.9 (36.1)	187.6 (35.4)	< 0.0001
Triglyceride (mg/dL)	197.4 (118.6)	195.1 (114.4)	170.8 (101.1)	180.7 (117.7)	< 0.0001
nPCR	0.1 (0.3)	0.1 (0.3)	0.1 (0.3)	0.1 (0.3)	0.6788
Albumin (g/dL)	3.8 (0.4)	3.8 (0.3)	3.8 (1.2)	3.7 (0.4)	0.0355
ESA dose, (U/month)	16,238.0 (10,760.4)	16,718.5 (6293.3)	16,390.6 (6790.4)	16,741.5 (9510.2)	0.4160
IV Iron, n (%)	219 (50.2)	1018 (53.8)	807 (44.2)	37 (18.5)	< 0.0001

ESA: erythropoiesis-stimulating agent; eGFR: estimated glomerular filtration rate; IV: intravenous; nPCR: normalized protein catabolic rate; PTH, parathyroid hormone; TSAT: transferrin saturation; WBC: white blood cell counts. All values are expressed as the mean (SD) unless otherwise specified.

### Associations of hemoglobin with mortality in PD patients

**Figure 2:**
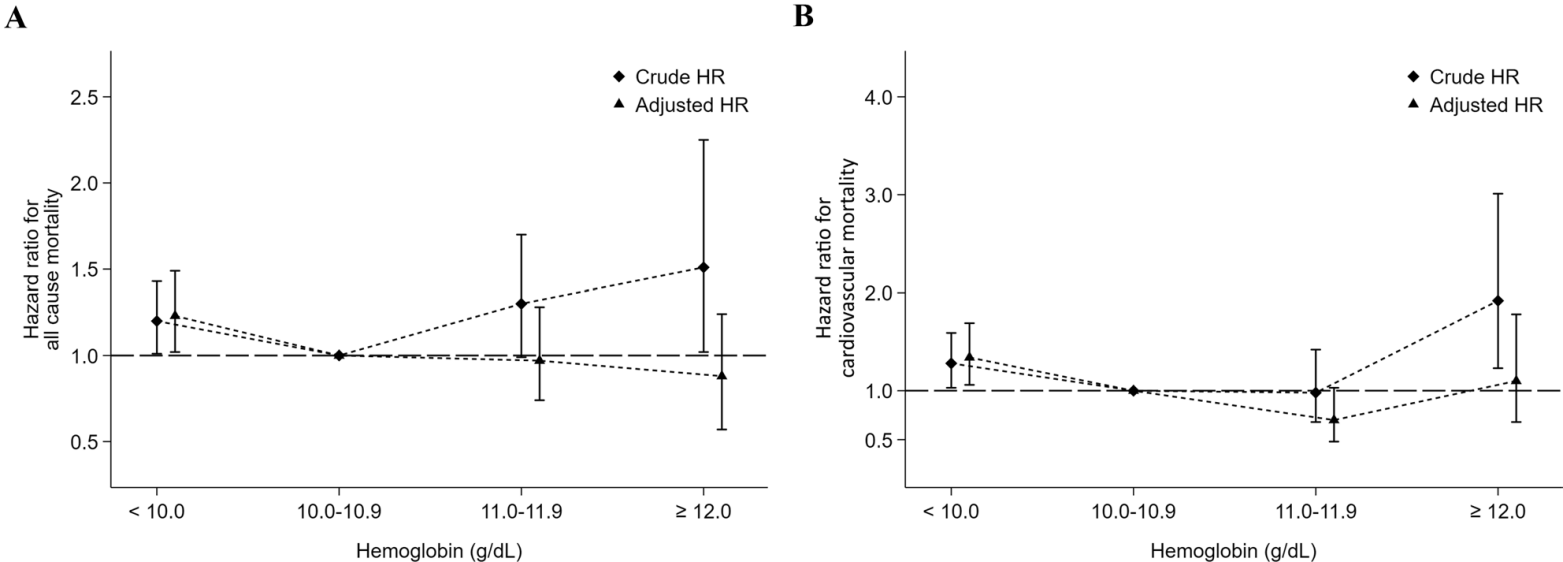
Associations between hemoglobin values and all-cause (**A**), cardiovascular (**B**) mortality, respectively, in 4356 maintenance peritoneal dialysis patients.

During a median follow-up of 2.9 years (maximum 7.9 years), 694 (15.9%) patients died. Figure 2 shows the crude and adjusted hazard ratios (aHRs) for mortality among the patients of the four hemoglobin categories. In a multivariate Cox proportional hazard model, for PD patients with a hemoglobin < 10 g/dL, the aHRs were 1.23 (95% CI 1.02–1.49, p = 0.03) for all-cause mortality, 1.34 (95% CI 1.06–1.69, p = 0.013) for CV mortality, 0.69 (95% CI 0.36–1.33, p = 0.27) for infection-associated mortality and 1.39 (95% CI 0.66–2.92, p = 0.38) for cancer-associated mortality with respect to those with a hemoglobin of 10–10.9 g/dL. In contrast, for PD patients with a hemoglobin level ≥ 12 g/dL, the aHRs were 0.88 (95% CI 0.57–1.24, p = 0.54) for all-cause mortality, 1.10 (95% CI 0.68–1.78, p = 0.71) for CV mortality, and 0.42 (95% CI 0.05–3.38, p = 0.41) for infection-associated mortality with respect to those with a hemoglobin level within 10–10.9 g/dL (Table 4). The results were similar for all ESA-treated PD patients (Supplemental Table S2).

**Table 4:** Hemoglobin value, iron parameters and the risks of all-cause, cardiovascular, infection-related and cancer-related mortality among peritoneal dialysis patients.

	Events	IR	cHR	aHR
**Hemoglobin (g/dL)**^1^				
All-cause mortality				
< 10	407	50.36	1.20 (1.01–1.43), p = 0.038	1.23 (1.02–1.49), p = 0.03
10–10.9	182	39.79	1.0 (reference)	1.0 (reference)
11–11.9	77	48.19	1.30 (1.00–1.70), p = 0.05	0.97 (0.74–1.28), p = 0.85
≥ 12	28	57.80	1.51 (1.02–2.25), p = 0.042	0.88 (0.57–1.24), p = 0.54
Cardiovascular mortality				
< 10	278	34.40	1.28 (1.03–1.59), p = 0.025	1.34 (1.06–1.69), p = 0.013
10–10.9	118	25.80	1.0 (reference)	1.0 (reference)
11–11.9	38	23.78	0.98 (0.68–1.42), p = 0.93	0.70 (0.48–1.03), p = 0.07
≥ 12	23	47.48	1.92 (1.23–3.01), p = 0.001	1.10 (0.68–1.78), p = 0.71
Infection-related mortality				
< 10	28	3.46	0.82 (0.45–1.48), p = 0.51	0.69 (0.36–1.33), p = 0.27
10–10.9	18	3.94	1.0 (reference)	1.0 (reference)
11–11.9	14	8.76	2.43 (1.21–4.89), p = 0.013	1.96 (0.94–4.09), p = 0.07
≥ 12	1	2.06	0.55 (0.07–4.14), p = 0.56	0.42 (0.05–3.38), p = 0.41
Cancer-related mortality				
< 10	30	3.71	1.32 (0.67–2.58), p = 0.42	1.39 (0.66–2.92), p = 0.38
10–10.9	12	2.62	1.0 (reference)	1.0 (reference)
11–11.9	4	2.50	1.05 (0.34–3.27), p = 0.93	0.74 (0.23–2.44), p = 0.62
≥ 12	–	–	–	–
**Ferritin (ng/mL)**^**2**^				
All-cause mortality				
< 300	233	36.63	1.00 (0.81–1.22), p = 0.97	0.94 (0.76–1.16), p = 0.56
300–499	148	37.55	1.0 (reference)	1.0 (reference)
500–799	154	56.84	1.52 (1.21–1.90), p = 0.001	1.08 (0.85–1.37), p = 0.51
≥ 800	159	92.06	2.50 (2.00–3.13), p = 0.001	1.31 (1.02–1.70), p = 0.036
Cardiovascular mortality				
< 300	158	24.84	1.03 (0.80–1.33), p = 0.80	1.01 (0.77–1.31), p = 0.95
300–499	97	24.61	1.0 (reference)	1.0 (reference)
500–799	105	38.76	1.58 (1.20–2.09), p = 0.001	1.11 (0.83–1.48), p = 0.49
≥ 800	97	56.16	2.33 (1.76–3.08), p = 0.001	1.16 (0.84–1.59), p = 0.37
Infection-related mortality				
< 300	17	2.67	0.67 (0.34–1.33), p = 0.25	0.56 (0.27–1.17), p = 0.12
300–499	16	4.06	1.0 (reference)	1.0 (reference)
500–799	13	4.80	1.18 (0.57–2.45), p = 0.66	0.98 (0.45–2.13), p = 0.96
≥ 800	15	8.69	2.19 (1.08–4.43), p = 0.029	1.41 (0.59–3.34), p = 0.44
Cancer-related mortality				
< 300	12	1.89	0.76 (0.33–1.75), p = 0.52	0.71 (0.30–1.70), p = 0.44
300–499	10	2.54	1.0 (reference)	1.0 (reference)
500–799	7	2.58	1.01 (0.39–2.66), p = 0.98	0.60 (0.22–1.68), p = 0.33
≥ 800	17	9.84	3.98 (1.82–8.69), p = 0.001	1.78 (0.71–4.51), p = 0.22
**TSAT (%)**^**3**^				
All-cause mortality				
< 20	82	61.69	1.46 (1.14–1.86), p = 0.003	1.35 (1.02–1.78), p = 0.035
20–29	263	40.07	0.90 (0.76–1.06), p = 0.21	1.01 (0.84–1.22), p = 0.90
30–49	277	44.49	1.0 (reference)	1.0 (reference)
≥ 50	72	116.27	2.72 (2.10–3.53), p = 0.001	1.60 (1.21–2.11), p = 0.001
Cardiovascular mortality				
< 20	54	40.63	1.45 (1.07–1.96), p = 0.017	1.25 (0.89–1.77), p = 0.20
20–29	181	27.58	0.93 (0.76–1.15), p = 0.51	0.99 (0.79–1.24), p = 0.95
30–49	184	29.55	1.0 (reference)	1.0 (reference)
≥ 50	38	61.36	2.18 (1.54–3.09), p = 0.001	1.25 (0.86–1.81), p = 0.25
Infection-related mortality				
< 20	7	5.27	1.66 (0.70–3.90), p = 0.25	2.11 (0.78–5.73), p = 0.14
20–29	24	3.66	1.08 (0.60–1.94), p = 0.80	1.56 (0.81–2.99), p = 0.18
30–49	21	3.37	1.0 (reference)	1.0 (reference)
≥ 50	9	14.53	4.52 (2.07–9.88), p = 0.001	2.83 (1.16–6.87), p = 0.022
Cancer-related mortality				
< 20	4	3.01	1.34 (0.44–4.03), p = 0.60	1.18 (0.34–4.11), p = 0.80
20–29	15	2.29	0.94 (0.46–1.93), p = 0.88	1.13 (0.52–2.48), p = 0.76
30–49	15	2.41	1.0 (reference)	1.0 (reference)
≥ 50	12	19.38	8.31 (3.88–17.8), p = 0.001	4.15 (1.77–9.75), p = 0.01

aHR: adjusted hazard ratio; cHR: crude hazard ratio; IR: incidence rate per 1000 patient-years.

^1^aHRs were adjusted for age, sex, diabetes, hypertension, dialysis adequacy [weekly Kt/V, and weekly creatinine clearance (weekly CCr)], estimated glomerular filtration rate (eGFR) at the start of dialysis (MDRD), white blood cell counts, the normalized protein catabolic rate (nPCR), serum albumin, cholesterol, triglyceride, ferritin, transferrin saturation, calcium, phosphate, alkaline phosphatase, intact-PTH, uric acid, erythropoiesis-stimulating agents dose, and intravenous iron use.

^2^aHRs were adjusted for age, sex, diabetes, hypertension, dialysis adequacy (weekly Kt/V and weekly CCr), estimated glomerular filtration rate (eGFR) at the start of dialysis (MDRD), white blood cell counts, the normalized protein catabolic rate (nPCR), serum albumin, cholesterol, triglyceride, hemoglobin, transferrin saturation, calcium, phosphate, alkaline phosphatase, intact-PTH, uric acid, erythropoiesis-stimulating agents dose, and intravenous iron use.

^3^aHRs were adjusted for age, sex, diabetes, hypertension, dialysis adequacy (weekly Kt/V and weekly CCr), estimated glomerular filtration rate (eGFR) at the start of dialysis (MDRD), white blood cell counts, the normalized protein catabolic rate (nPCR), serum albumin, cholesterol, triglyceride, ferritin, hemoglobin, calcium, phosphate, alkaline phosphatase, intact-PTH, uric acid, erythropoiesis-stimulating agents dose, and intravenous iron use.

### Associations of iron parameters with mortality in PD patients

**Figure 3:**
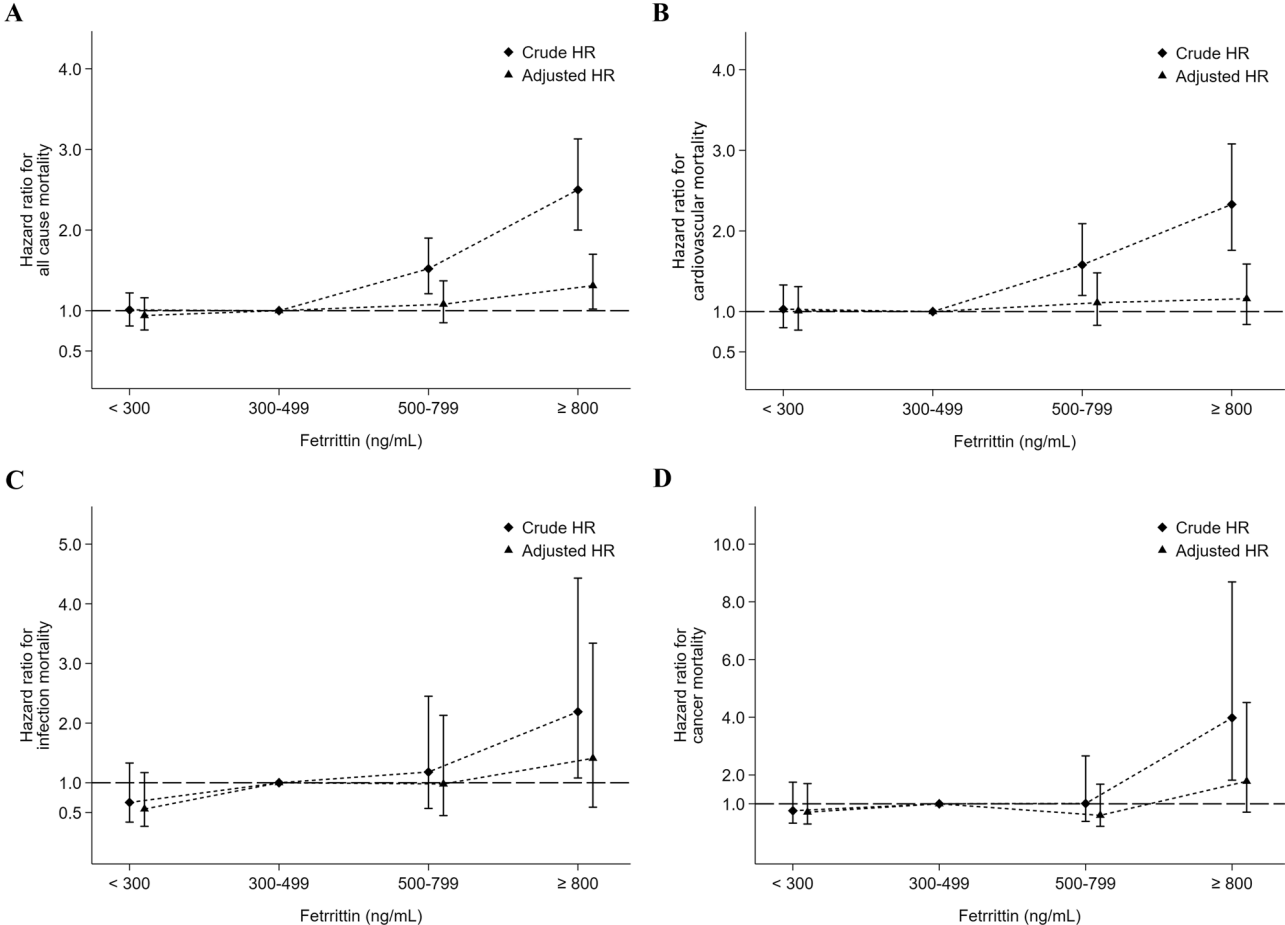
Associations between serum ferritin levels and all-cause (**A**), cardiovascular (**B**), infection-related (**C**) and cancer-related (**D**) mortality, respectively, in 4356 maintenance peritoneal dialysis patients.

The associations of different ferritin categories with mortality were also evaluated, as shown in Fig. 3 and Table 4. In a multivariate Cox proportional hazard model, the aHRs were 0.94 (95% CI 0.76–1.16) for all-cause mortality, 1.01 (95% CI 0.77–1.31) for CV mortality, 0.56 (95% CI 0.27–1.17) for infection-related mortality and 0.71 (95% CI 0.30–1.70) for cancer-related mortality for patients with a serum ferritin level < 300 ng/mL with respect to those with ferritin levels between 300–499 ng/mL, but none of these were significant (Table 4). On the other hand, the crude HRs for all-cause mortality, CV mortality and infection-related mortality were significantly higher in those with a serum ferritin level ≥ 800 ng/mL. However, after multivariate adjustment, only the aHR for all-cause mortality (1.31; 95% CI 1.02–1.70) was significantly higher in those with a serum ferritin level ≥ 800 ng/mL.

**Figure 4:**
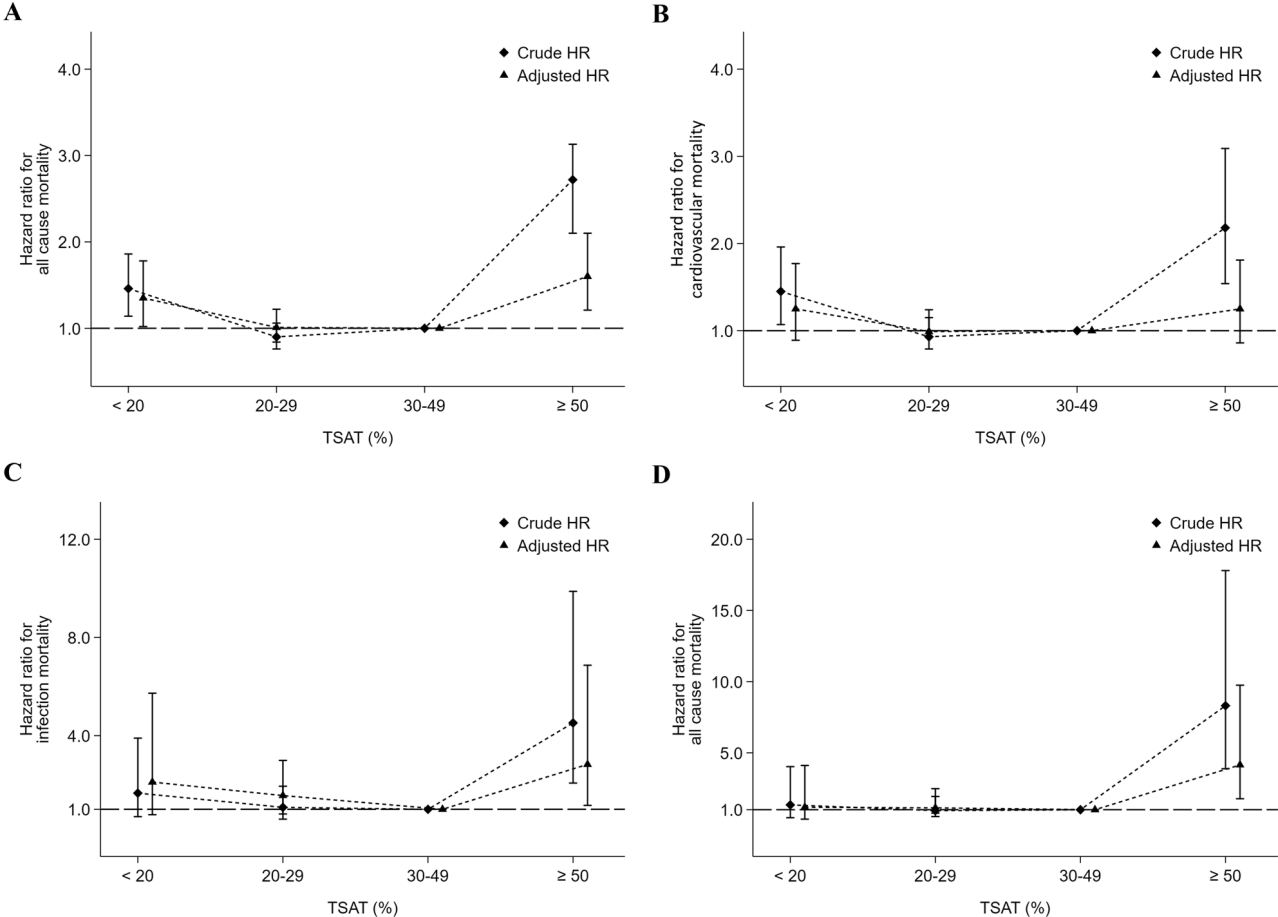
Associations between transferrin saturation (TSAT) values and all-cause (**A**), cardiovascular (**B**), infection-related (**C**) and cancer-related (**D**) mortality, respectively, in 4356 maintenance peritoneal dialysis patients.

Figure 4 and Table 4 show the associations between different TSAT categories and mortality. In a multivariate Cox proportional hazard model, the aHR for all-cause mortality (1.35; 95% CI 1.02–1.78) was significantly higher in those with a TSAT < 20% but modestly higher for CV mortality (1.25; 95% CI 0.89–1.77), infection-related mortality (2.11; 95% CI 0.78–5.73) and cancer mortality (1.18; 95% CI 0.34–4.11). On the other hand, the aHRs for all-cause (1.60; 95% CI 1.21–2.11), infection-related (2.83; 95% CI 1.16–6.87) and cancer (4.15; 95% CI 1.77–9.75) mortalities were significantly increased in those with a TSAT ≥ 50%. In addition, a TSAT value in a range of 20–50% was associated with a lower risk for mortality. Supplemental Figure S1 shows the cubic spline curves for the associations of hemoglobin, ferritin and TSAT with the risk of all-cause mortality. The findings showed similar trends, as shown in Figs. 2A, 3A and 4A.

### Associations of iron status and iron supplementation with the risk of death in PD patients

We further validated the association of iron status and iron supplementation with the risk of all-cause mortality. PD patients were divided into three groups: serum ferritin < 800 ng/mL, TSAT < 50% and receiving iron supplementation (Group 1, n = 1940), serum ferritin < 800 ng/mL, TSAT < 50% but not receiving iron supplementation (Group 2, n = 1803) and serum ferritin ≥ 800 ng/mL or TSAT ≥ 50% (Group 3, n = 613) (Supplemental Table S3). Compared with Group 1 patients, Group 2 and Group 3 patients were older and more likely to be male and had lower serum albumin but a higher dialysis inadequacy rate. Kaplan–Meier (Supplemental Figure S2) and Cox proportional hazard (Supplemental Figure S3) survival curves demonstrated that Group 2 and 3 patients were associated with a significantly higher risk for all-cause death than Group 1 patients. In a multivariate Cox proportional hazard model (Supplemental Table S4), compared with Group 1 patients (reference group), the aHRs for all-cause mortality were significantly higher in Group 2 (1.75; 95% CI 1.41–2.15) and 3 patients (2.54; 95% CI 2.01–3.20).

## Discussion

The prevalence of CKD in Taiwan, 11.9%, is very high^12^. According to data from the United States Renal Data System (USRDS), the incidence and prevalence of treated ESRD in Taiwan ranked first in the world from 2002 to 2016^13^. In Taiwan, anemia is a common problem in CKD patients. Wen et al*.* disclosed that 58.8% of stage 4 Taiwanese CKD patients are anemic, increasing to 92.5% in Taiwanese patients with stage 5 CKD^12^. Taiwan’s government launched the national health insurance (NHI) system on March 1, 1995, ensuring the right of healthcare for all citizens and offering free access to medical services and coverage of medical expenses for dialysis therapy. The NHI also implemented a fully bundled payment system for dialysis expenses, including the costs of dialysis, dialysis-associated laboratory tests, and the use of ESAs, iron supplements, calcium-containing phosphate binders, and vitamin D. Later, the NHI applied restrictive reimbursement criteria for ESA use targeting a lower hematocrit in patients with non-dialyzed stage 5 CKD or those under dialysis in 1996. ESAs are to be initiated when dialysis-dependent CKD patients have a hematocrit of < 28% in order to maintain a hematocrit of 30%. The maximum doses allowed by insurance is capped at 20,000 U of epoetin-α or β and 100 µg of NESP or Mircera per month. However, the conservative hematocrit target of 30% for stage 5 CKD patients set by the Taiwan NHI is related to economic rather than evidence-based concerns. Moreover, the suggestions are mainly for HD patients. The optimal values of hemoglobin, serum ferritin, and TSAT associated with a better survival rate in PD patients following the implementation of the bundled payment system in Taiwan thus deserve further assessment.

According to our previous studies^8,9^, the hemoglobin value for Taiwanese dialysis patients has been steady since 2000. Therefore, we analyzed the data from the TWRDS to investigate the associations of anemia and iron parameters with clinical outcomes in PD patients from 2001 to 2008. In addition, the blood hemoglobin concentration might change longitudinally in PD patients due to the presence of various disease statuses and treatment measures; measurement of a single hemoglobin level does not reflect an accurate assessment of a patient’s exposure to the effects of anemia management over time^14^. To address this problem, we analyzed the time-averaged hemoglobin value from each patient to validate the longitudinal burden of anemia by averaging all individual measurements and considering the duration of any individual value^14^. Our results demonstrated that a lower hemoglobin level was associated with significantly higher all-cause and CV mortality in PD patients; with a hemoglobin level of 10.0 to 10.9 g/dL as a reference, the adjusted death hazard ratios for hemoglobin levels of < 10 g/dL were 1.23 (95% CI 1.02–1.49) and 1.34 (95% CI 1.06–1.69), respectively. In contrast, the effect of normalized hemoglobin level on outcome was modest. The aHRs of all-cause and CV mortality for a hemoglobin level ≥ 12.0 g/dL were 0.88 (95% CI 0.57–1.24) and 1.10 (95% CI 0.68–1.78), respectively. The results of our study were similar to those of the study by Molnar et al*.* on PD patients^15^.

The influence of the fully bundled payment in anemia management was the use of iron, especially the IV form. In 1996, nephrologists in Taiwan reached a consensus regarding the diagnostic criteria for iron deficiency. They recommended that iron should be supplemented for dialysis patients with a ferritin level < 300 ng/mL and/or a TSAT level < 30% in order to maintain a ferritin level of 300–500 ng/mL and a TSAT value of 30–50%. The consensus was mainly based on our previous studies in HD patients performed in Taiwan, which provided guidance on the use of IV iron to correct renal anemia^16–19^. However, PD is a different modality of renal replacement therapy from HD, and whether these recommendations for iron supplementation and iron parameters on patient outcomes are suitable for PD patients is still unknown. Moreover, evidence about iron supplementation and iron parameters on PD patient outcomes has rarely been reported. Only one study from the Japanese nationwide dialysis registry, published by Maruyama et al., found that there were no clear associations between serum ferritin levels and mortality among PD patients^20^. To the best of our knowledge, our AIM-PD study is the first to report the association of optimal hemoglobin, serum ferritin, and TSAT values with mortality in PD patients. We demonstrated that those with a serum ferritin level ≥ 800 ng/mL were associated with an increased mortality. The safe upper limit of serum ferritin was similar to our previous report in patients predominantly receiving HD^9^. Fortunately, for accrediting PD units, the Taiwan Society of Nephrology has suggested that iron should not be supplemented when serum ferritin exceeds 800 ng/mL since 2005^8^. Therefore, the proportion of PD patients with a serum ferritin level > 800 ng/mL gradually decreased from 1995 to 2012. The recommendation of the 800 ng/mL threshold as the upper limit for serum ferritin during iron supplementation in Taiwanese PD patients could be based on epidemiological evidence according to our results. Accordingly, our study revealed that a TSAT value between 20 and 50% was associated with the lowest all-cause mortality. The results are in line with the recommendations by the 1996 Taiwan practice guidelines^8^ and the 2012 Kidney Disease: Improving Global Outcomes (KDIGO) clinical practice guidelines for iron management in CKD^21^. Finally, the AIM-PD study revealed that higher concentrations of serum TSAT (circulating iron) rather than ferritin (storage iron) were associated with increased risks of cancer death. This indicates that serum TSAT is a more useful biomarker of cancer risk than serum ferritin in PD patients. This phenomenon is similar to the observational study proposed by Chua et al. from the Busselton Health Survey^22^.

From a clinical perspective, several points from the AIM-PD study deserved further discussion. Our study is noteworthy for its large sample size, nationally representative, and the study cohort was validated by Taiwanese NHI reimbursement regulations^8,9^. Despite this, some issues should be discussed in AIM-PD study. First, our study is observational and cannot prove causality. To test the hypothesis that anemia decreased survival by affecting cardiovascular factors, we found that the effect of anemia on all-cause mortality was similar to the trend for CV mortality^9^. Second, the therapeutic effects of ESAs could not be directly measured. We demonstrated that a lower time-averaged hemoglobin was associated with higher all-cause and CV mortality for all ESA-treated PD patients (Supplemental Table S2), and the trend was also similar for all PD patients (Table 4), indicating the protective effects of ESA use in PD patients. The result is similar as our study in Taiwanese HD patients^9^. Third, although the inflammatory marker of C-reactive protein was not available in the AIM-PD study, we excluded poorly nourished patients and used serum albumin, TSAT, ferritin and white blood cell data, which are associated with the inflammation-malnutrition complex, to adjust for potential bias^9^. Fourth, we recruited well-nourished and excluded patients younger than 20 years and those who died or could not undergo a follow-up within one year after the initiation of HD. Our cohort could not represent the entire PD population. Finally, in addition to different anemia management strategy, the administrated IV iron preparations in our cohort are mainly traditional formulations such as iron sucrose and ferric chloride hexahydrate which differ from other countries^23–25^. Therefore, our data are not generalizable to populations outside Taiwan. Fortunately, recent studies from FERWON-NEPHRO trials^26,27^ and results from PIVOTAL (Proactive IV Iron Therapy in Haemodialysis Patients) trials^28,29^ demonstrated that application of new iron formulation associated with fewer cardiovascular adverse effect and proactive iron therapy provided better clinical outcome. These results may change our anemia management policy in the future.

In summary, a lower hemoglobin value (less than 10 g/dL) was associated with a higher risk of all-cause and CV death among PD patients receiving inadequately low administered ESA doses in the bundled payment system. The result is similar as our previous study in HD patients^9^. In addition, a serum ferritin level > 800 ng/mL was associated with all-cause mortality. A TSAT between 20 and 50% was associated with a lower risk for all-cause mortality. Therefore, we recommend avoiding a low hemoglobin value and a serum ferritin more than 800 ng/mL and maintaining a TSAT between 20–50% in prevalent PD patients receiving restricted ESA doses but prompt iron supplementation based on the findings of the AIM-PD study.

## Methods

### Data source

The data and study materials will not be made available to other researchers for purposes of reproducing the results or replicating the procedure because access to these data is contractually controlled by the Taiwan Society of Nephrology and Taiwan National Health Research Institutes. Only analytic methods are available on request. A request for the analytic methods should be sent to the corresponding author.

The TWRDS integrates the records of all end-stage renal disease (ESRD) patients requiring HD and PD from hospitals and dialysis clinics in Taiwan^10,30^. The TWRDS database includes the demographic characteristics, disease-associated conditions, initial dialysis date, dialysis type, residual renal function, and laboratory data of each dialysis patient in Taiwan. Annual reports from dialysis facilities, including dialysis dosages, treatment quality, laboratory data and clinical outcomes, are collected. Since 1997, the percentage of reports received from hospitals and dialysis centers each year has been 100%^10,30^. Informed consent is waived due to personal information that had been de-identified in the TWRDS Database and informed consent waiver was approved by Institutional Review Board of Taipei Veterans General Hospital, Taiwan (IRB –TPEVGH No.: 2016-12-009BC). The AIM-PD study was carried out in accordance with the approved protocol and the Declaration of Helsinki. All experimental protocols were approved by the Institutional Review Board of Taipei Veterans General Hospital, Taipei, Taiwan.

### Design and study participants

We conducted a cohort study based on the TWRDS and identified all incident ESRD patients in Taiwan from January 1, 2001, to June 30, 2008. The details of this cohort were described in our previous study^9,31^. The exclusion criteria included patients treated by hemodialysis, recipients of kidney transplantation, those with incomplete biochemistry data and those younger than 20 years of age when dialysis was initiated. All enrolled patients were divided into subgroups according to the time-averaged hemoglobin, serum ferritin and TSAT levels and were followed up until death or December 31, 2008, whichever came first. Mortality records were retrieved from the Taiwan Death Registry at the Taiwan Ministry of Health and Welfare. The outcomes included all-cause mortality, cardiovascular mortality, and mortality due to infection and cancer.

### Statistical analysis

All values are expressed as the means and SDs unless otherwise specified. The baseline characteristics were compared by analysis of variance (ANOVA) or chi-square tests. In a multivariate Cox regression model, the effects of hemoglobin, ferritin, and TSAT values were adjusted for age, sex, diabetes mellitus, hypertension, dialysis adequacy [weekly Kt/V, weekly creatinine clearance (weekly CCr)], residual renal function by estimated glomerular filtration rate (GFR) at the start of dialysis (MDRD), white blood cell count, normalized protein catabolic rate (nPCR), serum albumin, cholesterol, triglycerides, calcium, phosphate, alkaline phosphatase, intact parathyroid hormone, uric acid, ESA dose, and IV iron use. We used the log-minus-log plot and Schoenfeld residuals for a non-zero slope plot to test the Cox PH proportionality HR assumption (Supplemental Figure S4 and S5). The results are presented as Kaplan–Meier plots or expressed as HRs and 95% CIs. For incomplete cases in this study, we used the expectation–maximization algorithm to impute and replace each missing value. Restricted cubic spline curves were used to examine the nonlinear associations of hemoglobin, ferritin, and TSAT values with all-cause mortality for fitness, adjusted for the aforementioned confounding variables, which further characterized the nature of the relationships between these values and all-cause mortality. Five knots were chosen because this number produced a curve that appeared adequately smooth. Plots of the restricted splines were constructed using STATA version 15 (STATA Corp, College Station, TX). The variance inflation factor (VIF) was used in the collinearity analysis and the plot of Cox Snell residual and Nelsen-Aalen cumulative hazard were used in Goodness of fit analysis (Supplemental Figure S6). All p-values were two-sided, and the significance level was set at 0.05. All analyses, except for special circumstances, were performed using commercially available software (SAS, version 9.4, SAS Institute Inc., Cary, NC).

## Supplementary Information

Supplementary Information.
