# Supplementary material for: Association of anemia and iron parameters with mortality among prevalent peritoneal dialysis patients in Taiwan: the AIM-PD study
Source: Sci Rep. 2022 Jan 24;12:1269. doi: 10.1038/s41598-022-05200-3 (PMC8786856; doi:10.1038/s41598-022-05200-3)
Supplement: Supplementary file 1 — Supplementary Information. [file 41598_2022_5200_MOESM1_ESM.doc]

**Supplemental Data**

**Association of Anemia and Iron Parameters with Mortality Among Prevalent Peritoneal Patients in Taiwan: the AIM-PD Study**

Ko-Lin Kuo^1,2,3^, Jia-Sin Liu^4^, Ming-Huang Lin^5^, Chih-Cheng Hsu^5,6,7,8^, Der-Cherng Tarng^8,9,10,11^ and on behalf of the Taiwan Society of Nephrology Renal Registry Data System

^1^Division of Nephrology, Taipei Tzu Chi Hospital, Buddhist Tzu Chi Medical Foundation; ^2^School of Medicine, Tzu Chi University, Hualien; ^3^School of Post-Baccalaureate Chinese Medicine, Tzu Chi University, Hualien; ^4^Department of Public Health, Kaohsiung Medical University; ^5^Institute of Population Health Sciences, National Health Research Institutes, Taiwan; ^6^Department of Health Services Administration, China Medical University, Taichung, Taiwan; ^7^Department of Family Medicine, Min-Sheng General Hospital, Taoyuan, Taiwan; ^8^Institute of Clinical Medicine, National Yang Ming Chiao Tung University, Taipei, Taiwan; ^9^Center for Intelligent Drug Systems and Smart Bio-devices (IDS^2^B), Hsinchu, Taiwan; ^10^Department and Institute of Physiology, National Yang Ming Chiao Tung University, Taipei, Taiwan; ^11^Division of Nephrology, Department of Medicine, Taipei Veterans General Hospital, Taiwan

**Taiwan Society of Nephrology Renal Registry Data System Research Group**

Szu-Chun Hung^1^, Ko-Lin Kuo^1,2,3^, Jia-Sin Liu^4^, Chih-Cheng Hsu^5,6,7,8^, Ming-Huang Lin^5^, Der-Cherng Tarng^8,9,10,11^, Wei-Cheng Tseng^11^, Ming-Tsun Tsai^11^, Shuo-Ming Ou^11^, Chih-Yu Yang^11^, Yao-Ping Lin^11^, Yi-Sheng Lin^12^, Chia-Lin Wu^13^ & Tung-Po Hung^14^

^1^Division of Nephrology, Taipei Tzu Chi Hospital, Buddhist Tzu Chi Medical Foundation, Taipei, Taiwan; ^2^School of Medicine, Tzu Chi University, Hualien, Taiwan; ^3^School of Post-Baccalaureate Chinese Medicine, Tzu Chi University, Hualien, Taiwan; ^4^Department of Public Health, Kaohsiung Medical University, Kaohsiung, Taiwan; ^5^Institute of Population Health Sciences, National Health Research Institutes, Zhunan, Taiwan; ^6^Department of Health Services Administration, China Medical University, Taichung, Taiwan; ^7^Department of Family Medicine, Min-Sheng General Hospital, Taoyuan, Taiwan; ^8^Institute of Clinical Medicine, National Yang Ming Chiao Tung University, Taipei, Taiwan; ^9^Center for Intelligent Drug Systems and Smart Bio-devices (IDS^2^B), Hsinchu, Taiwan; ^10^Department and Institute of Physiology, National Yang Ming Chiao Tung University, Taipei, Taiwan; ^11^Division of Nephrology, Department of Medicine, Taipei Veterans General Hospital, Taiwan; ^12^Division of Nephrology, Taipei City Hospital, Taipei, Taiwan; ^13^Division of Nephrology, Changhua Christian Hospital, Changhua, Taiwan; ^14^Division of Nephrology, Wei Gong Memorial Hospital, Miaoli, Taiwan.

**Corresponding Author**

Chih-Cheng Hsu, MD, DrPH

Institute of Population Health Sciences, National Health Research Institutes, and Institute of Clinical Medicine, National Yang Ming Chiao Tung University, Taipei, Taiwan

35, Keyan Road, Zhunan Town, Miaoli County 35053, Taiwan

Tel.: 886-37-246166 ext 36336

Fax: 886-37-586261

Email: [cch@nhri.org.tw](mailto:cch@nhri.org.tw)

and

Der-Cherng Tarng, MD, PhD

Department and Institute of Physiology, National Yang Ming Chiao Tung University, and Division of Nephrology, Department of Medicine, Taipei Veterans General Hospital

201, Section 2, Shih-Pai Road, Taipei 11217, Taiwan

Phone: 886-2-2871 2121 ext. 2678

Fax: 886-2-28262837

Email: [dctarng@vghtpe.gov.tw](mailto:dctarng@vghtpe.gov.tw)

**Supplemental Figure S1**. Cubic spline and 95% confidence level of all-cause mortality for hemoglobin (A), serum ferritin (B) and transferrin saturation (TSAT) (C) in 4,356 maintenance peritoneal dialysis patients

**Supplemental Figure S2**. Kaplan–Meier analysis of survival curve among peritoneal dialysis patients from Taiwan Renal Registry Data System (2001–2008)

**Supplemental Figure S3**. Cox proportional hazard of survival curve among peritoneal dialysis patients from Taiwan Renal Registry Data System (2001–2008)

**Supplemental Figure S4**. The log-minus-log plot of hemoglobin (A), serum ferritin (B) and transferrin saturation (TSAT) (C) in peritoneal dialysis patients

**Supplemental Figure S5**. The non-zero slope of Schoenfeld residuals of h hemoglobin (A), serum ferritin (B) and transferrin saturation (TSAT) (C) in peritoneal dialysis patients

**Supplemental Figure S6**. The plot of Nelson-Aalen cumulative hazard function and the Cox-Snell residual

**Supplemental Table S1.** The comparison of anemia and iron parameters between TWRDS 2001-2008 and DOPPS 2020 in peritoneal dialysis patients

**Supplemental Table S2**. Associations of hemoglobin with all-cause, cardiovascular, infection-related and cancer-related mortality risks among peritoneal dialysis patients treated with erythropoiesis stimulating agents

**Supplemental Table S3.** Characteristics of peritoneal dialysis patients in different iron status by cut-off values of ferritin at 500 ng/mL and TSAT at 50% with or without iron supplementation

**Supplemental Table S4.** The risks of all-cause, cardiovascular, infection-related and cancer-related mortality among chronic peritoneal dialysis patients in different iron status by cut-off values of ferritin at 800 ng/mL and TSAT at 50% with or without iron supplementation.

**Supplemental Figure S1**

**
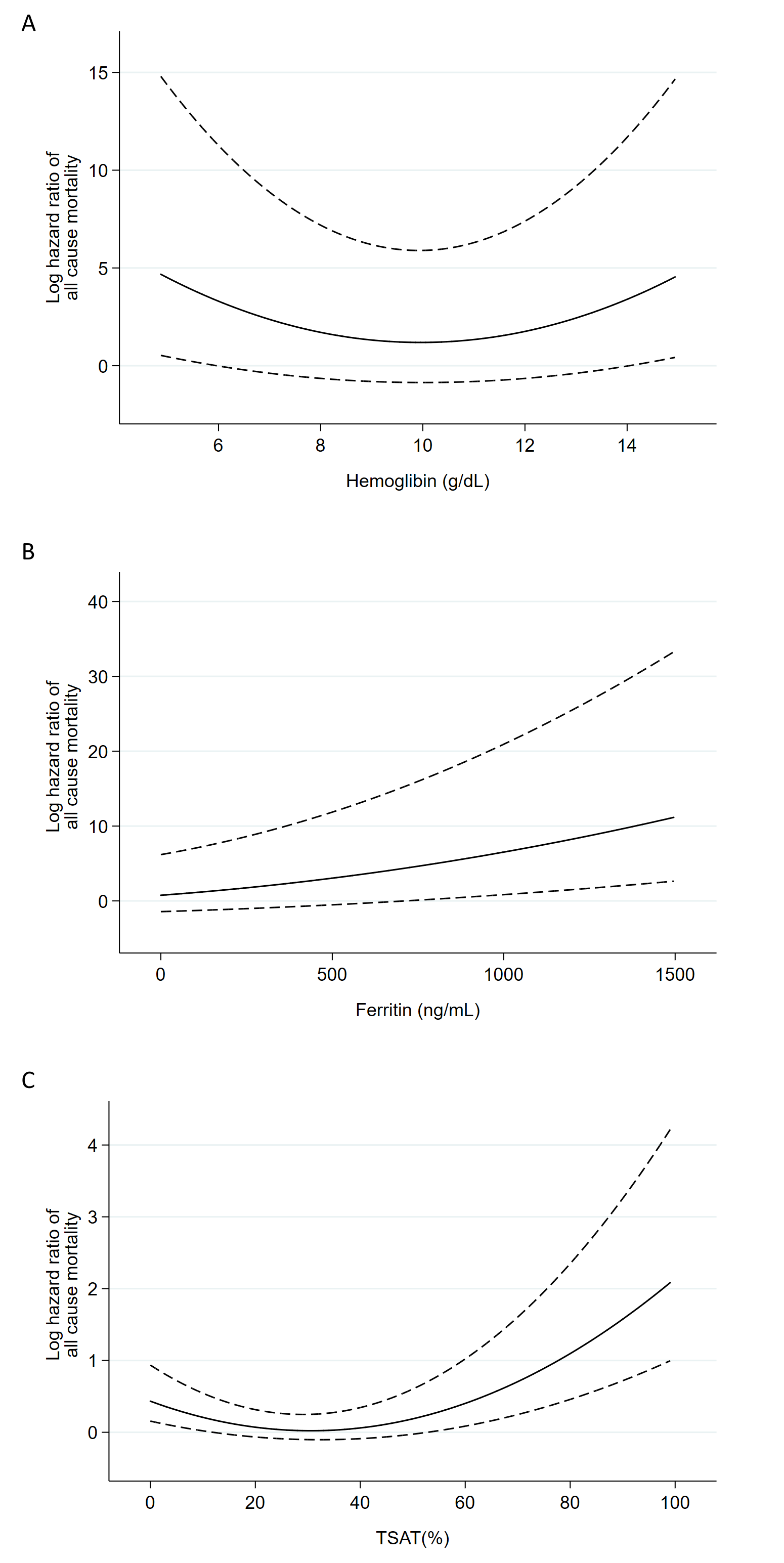
**

**Cubic spline and 95% confidence level of all-cause mortality for hemoglobin (A), serum ferritin (B) and transferrin saturation (TSAT) (C) in 4,356 maintenance peritoneal dialysis patients**

**Supplemental Figure S2**

**Kaplan–Meier analysis of survival curves among peritoneal dialysis patients from Taiwan Renal Registry Data System (2001–2008)**.

Group 1: ferritin < 800 ng/mL and TSAT < 50 % with iron supplementation,

Group 2: ferritin < 800 ng/mL and TSAT < 50 % without iron supplementation,

Group 3: ferritin ≥ 800 ng/mL or TSAT ≥ 50 %.

**Supplemental Figure S3**

**Cox proportional hazard of survival curves among peritoneal dialysis patients from Taiwan Renal Registry Data System (2001–2008)**.

Group 1: ferritin < 800 ng/mL and TSAT < 50 % with iron supplementation,

Group 2: ferritin < 800 ng/mL and TSAT < 50 % without iron supplementation,

Group 3: ferritin ≥ 800 ng/mL or TSAT ≥ 50 %.

**Supplemental Figure S4**


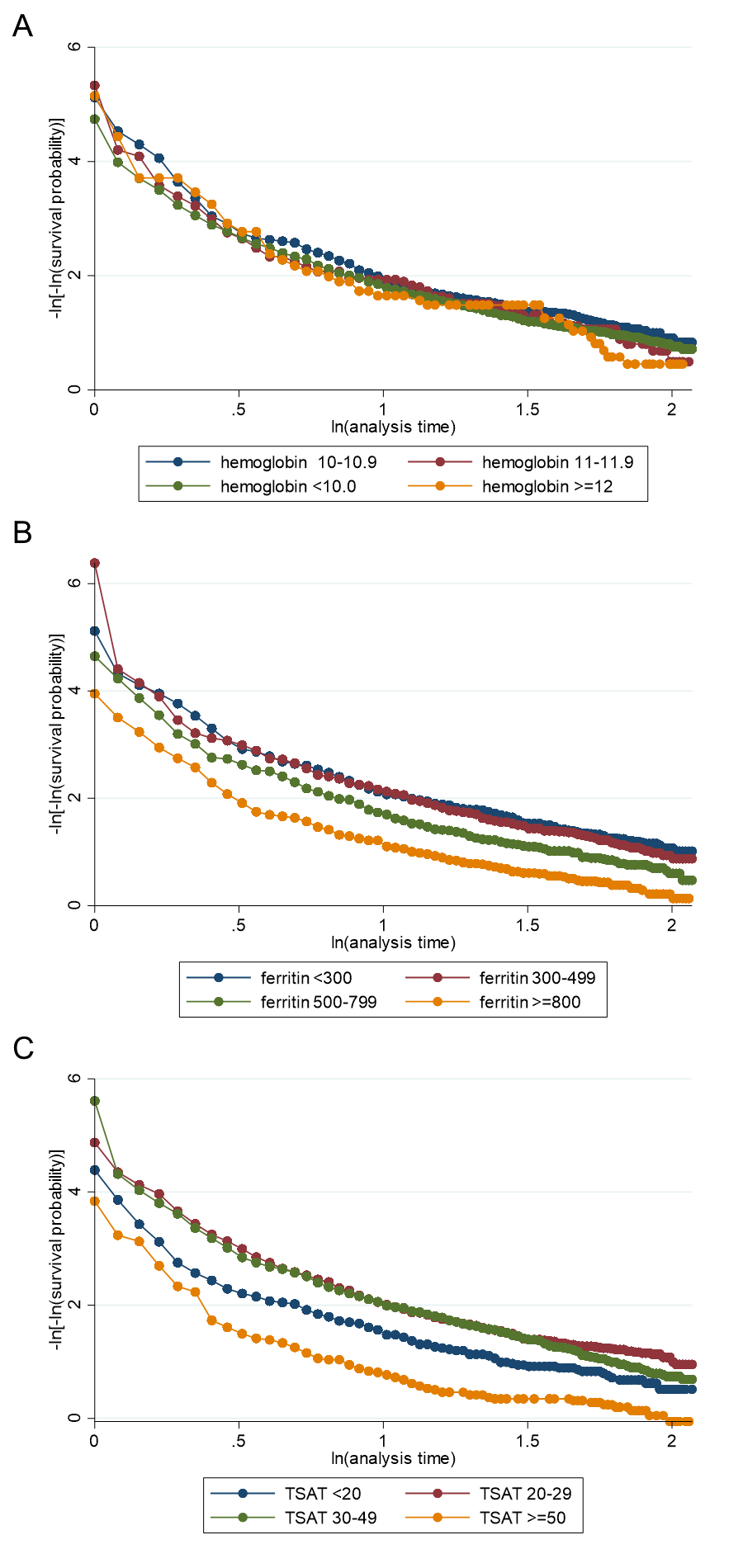


**The log-minus-log plot of hemoglobin (A), serum ferritin (B) and transferrin saturation (TSAT) (C) in peritoneal dialysis patients**

**Supplemental Figure S5**

**
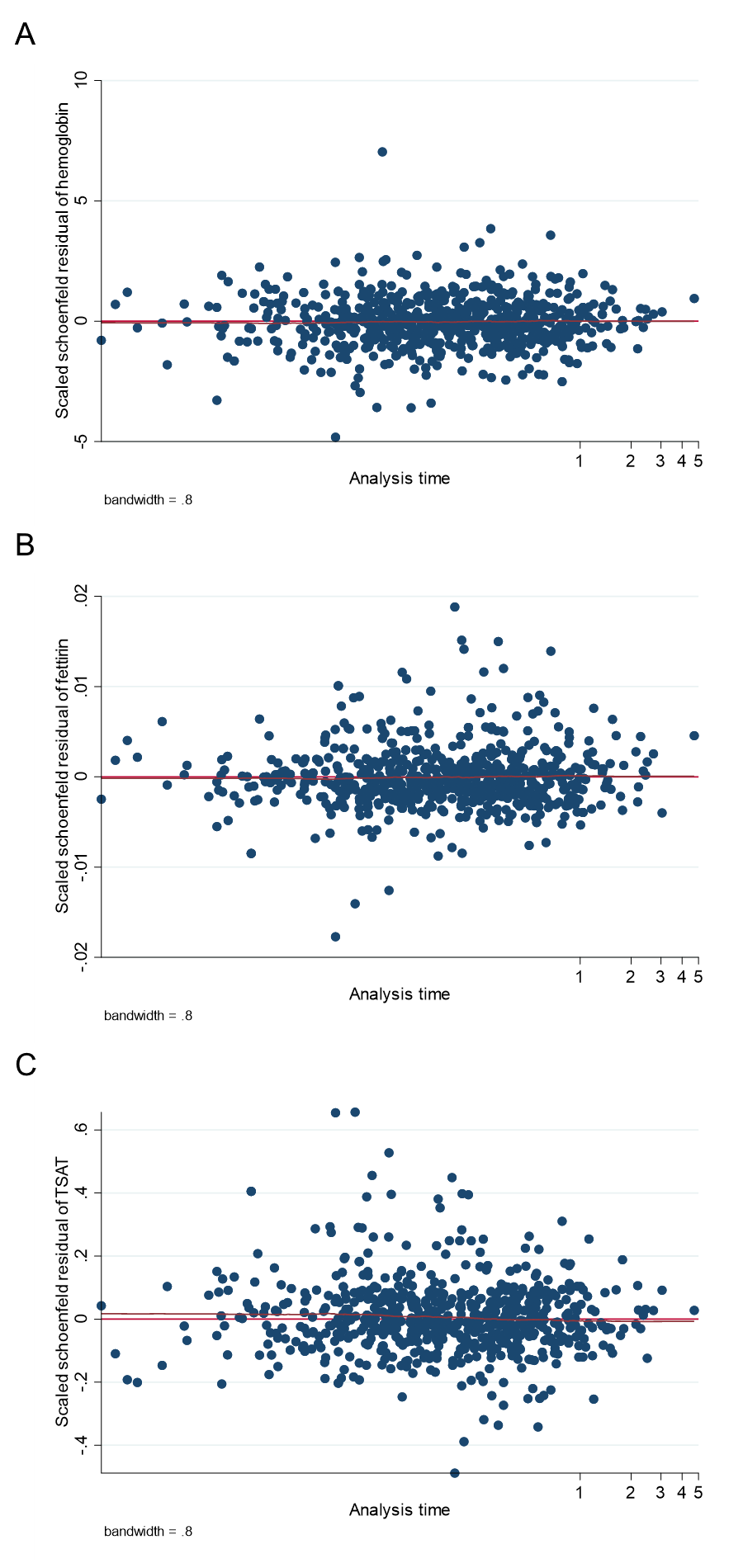
**

**The non-zero slope of Schoenfeld residuals of h hemoglobin (A), serum ferritin (B) and transferrin saturation (TSAT) (C) in** **peritoneal dialysis patients**

**Supplemental Figure S6**

**The plot of Nelson-Aalen cumulative hazard function and the Cox-Snell residual**

**Supplemental Table S1.** The comparison of anemia and iron parameters between TWRDS 2001-2008 and DOPPS 2020 in peritoneal dialysis patients

|  | **TWRDS 2001-2008** | **DOPPS in Nov. 2020** | P- value |
| --- | --- | --- | --- |
| **Hemoglobin (g/dL)** |  |  |  |
| n | 4338 | 3168 |  |
| n (%) |  |  | <0.001 |
| <10.0 | 2137 (49.3) | 687 (21.7) |  |
| 10-10.9 | 1445 (33.3) | 987 (31.2) |  |
| 11-11.9 | 598 (13.8) | 876 (27.7) |  |
| ≥12 | 165 (3.8) | 618 (19.5) |  |
| Mean (sd) | 10.0 (1.2) | 10.96 (2.3) | <0.001 |
|  |  |  |  |
| **Ferritin (mg/dL)** |  |  |  |
| n | 4338 | 3313 |  |
| n (%) |  |  | <0.001 |
| <499 | 3053 (70.4) | 1189 (35.9) |  |
| 500-799 | 778 (17.9) | 755 (22.8) |  |
| ≥800 | 525 (12.1) | 1369 (41.3) |  |
| Mean (sd) | 435 (369.0) | 732 (1153) | <0.001 |
|  |  |  |  |
| **TSAT (%)** |  |  |  |
| n | 4338 | 3326 |  |
| n (%) |  |  | <0.001 |
| <20 | 436 (10.1) | 491 (14.8) |  |
| 20-29 | 1892 (43.6) | 1228 (36.9) |  |
| 30-49 | 1828 (42.1) | 1364 (41.0) |  |
| ≥50 | 200 (4.6) | 243 (7.3) |  |
| Mean (sd) | 30.9 (10.7) | 30.3 (20.7) | 0.11 |

| **Supplemental Table S2.** Hemoglobin and all-cause, cardiovascular, infection-related and cancer-related mortality risks among peritoneal dialysis patients treated with erythropoiesis stimulating agents (n=3,132) | | | | |
| --- | --- | --- | --- | --- |
| Hemoglobin (g/dL) | Events | IR | cHR | aHR |
| All-cause mortality |  |  |  |  |
| < 10.0 | 373 | 48.29 | 1.21 (1.01-1.46), p= 0.04 | 1.30 (1.06-1.58), p= 0.01 |
| 10.0-10.9 | 162 | 37.41 | 1.0 (reference) | 1.0 (reference) |
| 11.0-11.9 | 56 | 38.43 | 1.11 (0.82-1.51), p= 0.48 | 0.91 (0.66-1.25), p= 0.55 |
| ≥12.0 | 17 | 48.85 | 1.39 (0.84-2.29), p= 0.20 | 0.68 (0.38-1.21), p= 0.19 |
| Cardiovascular mortality |  |  |  |  |
| < 10.0 | 252 | 32.63 | 1.29 (1.02-1.62), p= 0.031 | 1.41 (1.10-1.80), p= 0.006 |
| 10.0-10.9 | 104 | 24.01 | 1.0 (reference) | 1.0 (reference) |
| 11.0-11.9 | 27 | 18.53 | 0.83 (0.55-1.27), p= 0.40 | 0.67 (0.43-1.03), p= 0.07 |
| ≥12.0 | 16 | 45.98 | 2.04 (1.21-3.45), p= 0.008 | 1.07 (0.59-1.96), p= 0.82 |
| Infection-related mortality |  |  |  |  |
| < 10.0 | 26 | 3.37 | 0.88 (0.46-1.66), p= 0.69 | 0.80 (0.39-1.62), p= 0.53 |
| 10.0-10.9 | 15 | 3.46 | 1.0 (reference) | 1.0 (reference) |
| 11.0-11.9 | 11 | 7.55 | 2.42 (1.11-5.27), p= 0.026 | 2.26 (0.99-5.18), p= 0.053 |
| ≥12.0 | - | - | - | - |
| Cancer-related mortality |  |  |  |  |
| < 10.0 | 30 | 3.88 | 1.41 (0.71-2.82), p= 0.33 | 1.75 (0.79-3.85), p= 0.16 |
| 10.0-10.9 | 11 | 2.54 | 1.0 (reference) | 1.0 (reference) |
| 11.0-11.9 | 3 | 2.06 | 0.91 (0.25-3.26), p= 0.88 | 0.69 (0.18-2.68), p= 0.60 |
| ≥12.0 | - | - | - | - |

Abbreviations: aHR: adjusted hazard ratio; cHR: crude hazard ratio; IR: incidence rate per 1000 patient-years.

* aHRs were adjusted for adjusted for age, sex, diabetes, hypertension, dialysis adequacy (weekly Kt/V and weekly CCr), GFR at the start of dialysis (MDRD), white blood cell counts, the normalized protein catabolic rate (nPCR), serum albumin, cholesterol, triglyceride, hemoglobin, ferritin, transferrin saturation, calcium, phosphate, alkaline phosphatase, intact-PTH, uric acid, erythropoiesis-stimulating agents dose, and intravenous iron use.

**Cardiovascular mortality defined by ICD 9 codes 250, 261-263, 280-285, 410-414, 401-405, 440, 430-432, and 580-589. Infection-related mortality defined by ICD 9 codes 001-139, 420-429, 320-322, 326, 510-513, 567, 590, 599, 711, 730, 460-466, 480-487, 490-493, and 680-686. Cancer-related mortality defined by ICD 9 codes 140-208.

**Supplemental Table S3.** Characteristics of peritoneal dialysis patients in different iron status by cut-off values of ferritin at 500 ng/mL and TSAT at 50% with or without iron supplementation

|  | **Group 1 Group 2 Group 3** | | | |  |
| --- | --- | --- | --- | --- | --- |
| **Characteristics** | **Ferritin < 800 ng/mL and TSAT < 50 % with iron supplementation** | **Ferritin < 800 ng/mL and TSAT < 50 % without iron supplementation** | **Ferritin ≥ 800 ng/mL or TSAT ≥ 50 %** | ***P* value** |  |
| n | 1,940 | 1,803 | 613 |  |  |
| Age (years) | 49.3 (13.7) | 53.3 (14.9) | 54.8 (15.9) | <0.001 |  |
| Age group |  |  |  |  |  |
| 20-39 years, n (%) | 458 (23.6) | 343 (19) | 127 (20.7) | 0.003 |  |
| 40-64 years, n (%) | 1,201 (61.9) | 1,030 (57.1) | 295 (48.1) | <0.001 |  |
| 65-74 years, n (%) | 198 (10.2) | 279 (15.5) | 125 (20.4) | <0.001 |  |
| 75+ years, n (%) | 83 (4.3) | 151 (8.4) | 66 (10.8) | <0.001 |  |
| Gender |  |  |  |  |  |
| Female, n (%) | 1223 (63) | 899 (49.9) | 347 (56.6) | <0.001 |  |
| Diabetes, n (%) | 353 (18.2) | 504 (28) | 159 (25.9) | <0.001 |  |
| Hypertension, n (%) | 1,656 (85.4) | 1,106 (61.3) | 370 (60.4) | <0.001 |  |
| Weekly KT/V | 2.2 (0.3) | 2.1 (0.3) | 2.1 (0.3) | <0.001 |  |
| Weekly CCr (L/week/1.73 m^2^) | 61.4 (13.4) | 62.7 (14.6) | 58.6 (13.4) | <0.001 |  |
| eGFR at the start of dialysis (MDRD) | 4.7 (1.4) | 5.3 (2.7) | 5.4 (2.8) | <0.001 |  |
| WBC (× 10^3^ /μl) | 5.9 (2.3) | 6.2 (2.3) | 6.4 (2.4) | 0.014 |  |
| Hemoglobin (g/dL) | 10 (1) | 10.2 (1.2) | 9.5 (1.3) | <0.001 |  |
| Ferritin (ng/dL) | 310 (176) | 345 (201) | 1116 (472) | <0.001 |  |
| TSAT (%) | 28 (7) | 30 (8) | 44 (16) | <0.001 |  |
| Serum calcium (mg/dL) | 9.4 (0.6) | 9.3 (0.7) | 9.3 (0.7) | <0.001 |  |
| Serum phosphate (mg/dL) | 5.3 (0.9) | 5.1 (1.1) | 5.1 (1.2) | <0.001 |  |
| Alkaline phosphatase (U/L) | 102.3 (52.8) | 111.3 (59.5) | 116.4 (58.6) | <0.001 |  |
| Intact-PTH (pg/L) | 312 (252) | 276 (240) | 263 (133) | <0.001 |  |
| Uric acid (mg/dL) | 7 (1.1) | 7.1 (1.2) | 7 (1.2) | 0.32 |  |
| Cholesterol (mg/dL) | 201 (33) | 198 (37) | 198 (39) | 0.002 |  |
| Triglyceride (mg/dL) | 176 (102) | 184 (108) | 216 (133) | <0.001 |  |
| nPCR | 0.8 (0.5) | 0.6 (0.5) | 0.8 (0.5) | 0.14 |  |
| Albumin (g/dL) | 3.8 (0.3) | 3.8 (1.2) | 3.7 (0.4) | 0.002 |  |
| ESA dose, (U/month) | 17,948 (5,873) | 17,208 (8,203) | 18,381 (7,506) | <0.001 |  |
| ESA used (%) | 1,897 (97.8) | 1,600 (88.7) | 568 (92.7) | <0.001 |  |
| Iron IV, n (%) | 1,940 (45.5) | - | 141 (23.0) | <0.001 |  |

Abbreviations: ESA: erythropoiesis-stimulating agent; eGFR: estimated glomerular filtration rate; IV: intravenous; nPCR: normalized protein catabolic rate; PTH, parathyroid hormone; TSAT: transferrin saturation; WBC: white blood cell count

| **Supplemental Table S4.** The risks of all-cause, cardiovascular, infection-related and cancer-related mortality among chronic peritoneal dialysis patients in different iron status by cut-off values of ferritin at 800 ng/mL and TSAT at 50% with or without iron supplementation | | | | |
| --- | --- | --- | --- | --- |
| Hemoglobin (g/dL) | Events | IR | cHR | aHR |
| All-cause mortality |  |  |  |  |
| Group 1 | 144 | 20.61 | 1.0 (reference) | 1.0 (reference) |
| Group 2 | 360 | 62.66 | 3.19 (2.63-3.87), p=0.001 | 1.75 (1.41-2.15), p=0.001 |
| Group 3 | 190 | 94.63 | 4.75 (3.83-5.90), p=0.001 | 2.54 (2.01-3.20), p=0.001 |
| Cardiovascular mortality |  |  |  |  |
| Group 1 | 90 | 12.88 | 1.0 (reference) | 1.0 (reference) |
| Group 2 | 253 | 44.04 | 3.60 (2.83-4.58), p=0.001 | 1.91 (1.47-2.48), p=0.001 |
| Group 3 | 114 | 56.78 | 4.56 (3.46-6.02), p=0.001 | 2.26 (1.68-3.04), p=0.001 |
| Infection-related mortality |  |  |  |  |
| Group 1 | 16 | 2.29 | 1.0 (reference) | 1.0 (reference) |
| Group 2 | 26 | 4.53 | 2.08 (1.11-3.87), p= 0.021 | 0.89 (0.44-1.81), p= 0.75 |
| Group 3 | 19 | 9.46 | 4.30 (2.21-8.35), p=0.001 | 1.96 (0.94-4.10), p= 0.07 |
| Cancer-related mortality |  |  |  |  |
| Group 1 | 8 | 1.15 | 1.0 (reference) | 1.0 (reference) |
| Group 2 | 19 | 3.31 | 2.99 (1.31-6.84), p= 0.009 | 1.48 (0.60-3.67), p= 0.39 |
| Group 3 | 19 | 9.46 | 8.57 (3.75-19.6), p=0.001 | 4.56 (1.85-11.2), p=0.001 |

Abbreviations: IR: incidence rate per 1000 patient-years, cHR: crude hazard ratio; aHR: adjusted hazard ratio.

*aHRs were adjusted for adjusted for age, sex, diabetes, hypertension, dialysis adequacy (weekly Kt/V and weekly CCr), GFR at the start of dialysis (MDRD), white blood cell counts, the normalized protein catabolic rate (nPCR), serum albumin, cholesterol, triglyceride, hemoglobin, ferritin, transferrin saturation, calcium, phosphate, alkaline phosphatase, intact-PTH, uric acid, erythropoiesis-stimulating agents dose, and intravenous iron use.

**Cardiovascular mortality defined by ICD 9 codes 250, 261-263, 280-285, 410-414, 401-405, 440, 430-432, and 580-589. Infection-related mortality defined by ICD 9 codes 001-139, 420-429, 320-322, 326, 510-513, 567, 590, 599, 711, 730, 460-466, 480-487, 490-493, and 680-686. Cancer-related mortality defined by ICD 9 codes 140-208.

***Group definition:

Group 1: ferritin < 800 ng/mL and TSAT < 50 % with iron supplementation,

Group 2: ferritin < 800 ng/mL and TSAT < 50 % without iron supplementation,

Group 3: ferritin ≥ 800 ng/mL or TSAT ≥ 50 %
